# Identification of a Risk-Prediction Model for Hypertension Patients Concomitant with Nonalcoholic Fatty Liver Disease

**DOI:** 10.3390/healthcare13090969

**Published:** 2025-04-23

**Authors:** Xiaoyou Mai, Mingli Li, Xihui Jin, Shengzhu Huang, Mingjie Xu, Boteng Yan, Yushuang Wei, Xinyang Long, Yongxian Wu, Zengnan Mo

**Affiliations:** 1 School of Public Health, Guangxi Medical University, Nanning 530021, China; 202220926@sr.gxmu.edu.cn (X.M.); 202220934@sr.gxmu.edu.cn (Y.W.); 202210188@sr.gxmu.edu.cn (X.L.); 2 Center for Genomic and Personalized Medicine, Guangxi Key Laboratory for Genomic and Personalized Medicine, Guangxi Collaborative Innovation Center for Genomic and Personalized Medicine, Guangxi Medical University, Nanning 530021, China; limingligx@126.com (M.L.); 202220222@sr.gxmu.edu.cn (X.J.); hshzhu@sr.gxmu.edu.cn (S.H.); papapapa2025@163.com (M.X.); 202210111@sr.gxmu.edu.cn (B.Y.); wuyongxian@sr.gxmu.edu.cn (Y.W.); 3 Institute of Urology and Nephrology, First Affiliated Hospital of Guangxi Medical University, Guangxi Medical University, Nanning 530021, China

**Keywords:** NAFLD, HTN, risk factors, nomogram model

## Abstract

**Objective:** Our study aims to develop a personalized nomogram model for predicting the risk of nonalcoholic fatty liver disease (NAFLD) in hypertension (HTN) patients and further validate its effectiveness. **Methods:** A total of 1250 hypertensive (HTN) patients from Guangxi, China, were divided into a training group (875 patients, 70%) and a validation set (375 patients, 30%). LASSO regression, in combination with univariate and multivariate logistic regression analyses, was used to identify predictive factors associated with nonalcoholic fatty liver disease (NAFLD) in HTN patients within the training set. Subsequently, the performance of an NAFLD nomogram prediction model was evaluated in the separate validation group, including assessments of differentiation ability, calibration performance, and clinical applicability. This was carried out using receiver operating characteristic (ROC) curves, calibration curves, and decision curve analysis (DCA). **Results:** The risk-prediction model for the HTN patients concomitant with NAFLD included oral antidiabetic drugs (OADs) (OR = 2.553, 95% CI: 1.368–4.763), antihypertensives (AHs) (OR = 7.303, 95% CI: 4.168–12.794), body mass index (BMI) (OR = 1.145, 95% CI: 1.084–1.209), blood urea nitrogen (BUN) (OR = 0.924, 95% CI: 0.860–0.992), triglycerides (TGs) (OR = 1.474, 95% CI: 1.201–1.809), aspartate aminotransferase (AST) (OR = 1.061, 95% CI: 1.018–1.105), and AST/ALT ratio (AAR) (OR = 0.249, 95% CI: 0.121–0.514) as significant predictors. The AUC of the NAFLD risk-prediction model in the training set and the validation set were 0.816 (95% CI: 0.785–0.847) and 0.794 (95% CI: 0.746–0.842), respectively. The Hosmer–Lemeshow test showed that the model has a good goodness-of-fit (*p*-values were 0.612 and 0.221). DCA suggested the net benefit of using a nomogram to predict the risk of HTN patients concomitant with NAFLD is higher. These results suggested that the model showed moderate predictive ability and good calibration. **Conclusions:** BMI, OADs, AHs, BUN, TGs, AST, and AAR were independent influencing factors of HTN combined with NAFLD, and the risk prediction model constructed based on this could help to identify the high-risk group of HTN combined with NAFLD at an early stage and guide the development of interventions. Larger cohorts with multiethnic populations are essential to verify our findings.

## 1. Background

Nonalcoholic fatty liver disease (NAFLD) is a disease caused by excessive accumulation of triglycerides (TGs) in the liver due to metabolic dysfunction. It has become one of the fastest-growing causes of liver-related deaths worldwide and is projected to affect one-third of the global population by 2030 [1]. The disease spectrum of NAFLD includes nonalcoholic steatohepatitis (NASH) and its associated liver cirrhosis and hepatocellular carcinoma (HCC). Over the past 20 years, dramatic global lifestyle changes have exacerbated the NAFLD epidemic, making it a major chronic liver disease affecting the health of about 1.7 billion people worldwide [2]. NAFLD has become one of the most common liver diseases in China, but it has not received sufficient attention [3]. This disease is not only an important cause of disability and death from liver disease but also closely related to the metabolic syndrome, which is a major public health problem [4].

The global prevalence of NAFLD is 30.1% [3]. In addition, the prevalence of NAFLD in adults in high-income countries is at least 25–30%, and it is even higher, reaching 70–90%, in individuals with obesity or type 2 diabetes (T2DM) [5]. The latest meta-analysis shows that the prevalence of NAFLD in China has reached 29.2%, indicating a significant challenge in prevention and treatment [6]. In summary, as one of the most common causes of chronic liver disease, the high prevalence of NAFLD suggests that many patients may eventually develop serious liver health problems [7]. Therefore, the prevention and management of NAFLD is not only an individual health issue but also a global public health issue that requires urgent attention and focus.

Hypertension (HTN) is a clinical syndrome characterized primarily by persistently elevated systemic arterial blood pressure [8]. Its clinical diagnostic criteria are a systolic blood pressure (SBP) ≥ 140 mmHg or diastolic blood pressure (DBP) ≥ 90 mmHg [9], often accompanied by changes in the function or structure of organs such as the heart, brain, and kidneys. According to survey data from 2014 to 2017, the prevalence of HTN among individuals aged 35–75 in China is approximately 44.7% [10]. HTN results from the interaction of genetic predisposition and environmental risk factors and is a major cause of cardiovascular disease and early death worldwide, making it an important public health challenge [11].

NAFLD often coexists with HTN, as both are important components of the metabolic syndrome. According to recent epidemiological studies, approximately 50% of patients with HTN also have NAFLD [12]. In addition, the prevalence of HTN is significantly higher in NAFLD patients than in the general population [12,13,14] and the severity of NAFLD is closely related to the risk of developing HTN. 

HTN and NAFLD are both common chronic diseases, but relatively little research has been conducted on their co-existence. Considering their significant health risks and high treatment costs, early identification of at-risk populations and implementation of personalized interventions are crucial. We studied patients with hypertension combined with NAFLD with the aim of screening for independent influencing factors of HTN combined with NAFLD and constructing a column-line graph prediction model to facilitate the implementation of early interventions. We hope that by enhancing early prevention and control in high-risk groups, we can provide an important scientific basis for public healthcare, reduce the incidence of related diseases, and reduce the healthcare burden.

## 2. Methods

### 2.1. Study Design and Subjects

**Figure 1:**
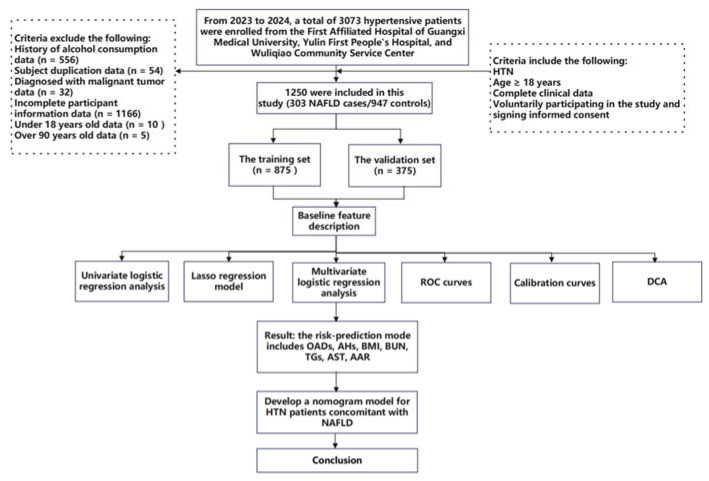
Flow diagram of the study design. NAFLD: nonalcoholic fatty liver disease; HTN: hypertension; ROC curves: receiver operating characteristic curves; and DCA: decision curve analyses.

This study is a cohort study combining both prospective and retrospective elements. The baseline survey was conducted at three medical centers in Nanning and Yulin cities, Guangxi Province, China, targeting patients from hospitals and community health service centers for extensive hypertension screening. From 1 October 2022 to 20 February 2024, participants were prospectively recruited for questionnaire surveys, clinical data collection, or blood sample collection for this study. All participants provided written informed consent at the time of recruitment. At the same time, retrospective electronic medical records of patients previously diagnosed with hypertension at these medical institutions were collected. During or after the data collection period, there was an opportunity to access information that could identify individual participants. A total of 3073 hypertensive patients admitted to the First Affiliated Hospital of Guangxi Medical University, the First People’s Hospital of Yulin, and Wuliqiao Community Health Service Center of Yulin between May 2023 and February 2024 were included in this study. The inclusion criteria are as follows: (1) patients met the diagnostic standards for HTN; (2) age ≥ 18 years; (3) voluntarily participating in the study and signing informed consent; (4) complete clinical data; (5) the patient had no recent stress such as infection, trauma, or surgery; and (6) the patient had no history of malignancy. The exclusion criteria are as follows: (1) age > 90 years; (2) concurrent liver diseases, such as drug-induced liver disease, viral hepatitis, autoimmune liver disease, or inherited metabolic liver disorders; (3) history of severe diseases, including psychiatric disorders, severe liver or kidney diseases, or malignant tumors; presence of immune or hematologic disorders or acute infectious diseases; (4) autoimmune diseases or immune deficiencies; (5) pregnancy or breastfeeding; and (6) use of interfering medications (e.g., antibiotics, immunosuppressants, or corticosteroids). Finally, 1250 hypertensive patients were considered as the target subjects, which included 303 NAFLD cases (HTN combined with NAFLD) and 947 non-NAFLD controls (HTN non-consolidated NAFLD). The study flowchart is shown in Figure 1. This study was conducted in accordance with the basic principles of the Declaration of Helsinki. This study received ethical approval from the Ethics and Human Subjects Committee of Guangxi Medical University, China (No. 2022-0193), and the Ethics and Human Subject Committee of First Affiliated Hospital of Guangxi Medical University, China (No. 2023-K090-01). 

### 2.2. Measurement of Indicators

The subjects were investigated by questionnaire, physical examination, and laboratory test. The questionnaire mainly includes general demographic characteristics (age, gender, ethnicity, occupational category, educational level, etc.), behavior and lifestyle (smoking, drinking, etc.), chronic medical history (HTN, T2DM, cardiovascular disease, stroke, etc.), and medication history. The physical examination indicators include height, weight, waist and hip circumference, blood pressure, etc. Fasting venous blood was collected for blood urea nitrogen (BUN), creatinine (Crea), total cholesterol (TC) and TGs, low-density lipoprotein cholesterol (HDL-C), low-density lipoprotein cholesterol (LDL-C), total bilirubin (TBIL), alanine aminotransferase (ALT), aspartate aminotransferase (AST), white blood cells (WBC), and other indicators. The laboratory test indexes were tested by the First People’s Hospital of Yulin and the First Affiliated Hospital of Guangxi Medical University.

### 2.3. Diagnostic Criteria

The clinical diagnostic criteria for HTN are as follows: in the absence of antihypertensive drugs, systolic blood pressure ≥ 140 mmHg and/or diastolic blood pressure ≥ 90 mmHg were measured 3 times on different days [9].

The diagnostic criteria for NAFLD are as follows: (1) liver near-field echo; diffuse enhancement (“bright liver”), echoes stronger than the kidneys; (2) the intrahepatic duct structure is not clear; (3) the far-field echo of the liver gradually attenuated. Those who meet the above criteria and who exclude factors such as alcohol intake, infection, or autoimmunity that may contribute to liver disease are classified as having NAFLD [15]. Body mass index (BMI) = weight (kg)/height^2^ (m)^2^. Smoking: defined as smoking an average of at least 1 cigarette/day for 6 months and being a current smoker. Alcohol consumption: drinking ≥ 1 times per week for ≥6 months.

### 2.4. Statistical Analyses

SPSS 26.0 and R software 4.3.3 were used for statistical analysis of the data. Continuous variables conforming to normal distribution were expressed as mean ± standard deviation ($\overset{-}{\chi }$ ± *s*), and comparison between the two groups was performed by *t*-test. Continuous variables with skewed distribution were expressed as the median (interquartile range), and the rank sum test was used for comparison between groups. Categorical variables were expressed as frequency (percentage), and the χ^2^ test was used for comparison between groups. The minimum absolute contraction and selection operator (LASSO) regression method was used to select the best predictors, and the selected factors were incorporated into the multi-factor logistic regression model. Based on the regression coefficients of independent variables, the nomogram prediction model was constructed using the rms package in R software. The area under the receiver operating characteristic (ROC) curve (AUC) was used to evaluate the predictive performance of the nomogram model. The bootstrap self-sampling method (*n* = 1000) was used to verify the model internally. The C-index, calibration curve, and Hosmer–Lemeshow test were used to evaluate the difference between model differentiation and calibration, with *p* < 0.05 as statistically significant. We evaluated the effectiveness of risk-prediction models in clinical decision-making by decision curve analyses (DCA).

## 3. Results

### 3.1. Demographic Characteristics of the Study Population

Of 1250 hypertensive subjects in the study, most of them were female (59.8%), of Han ethnicity (91.0%), non-smokers (88.7%), from Nanning sites (76.6%), and the median BMI was 24.2 (22.2, 26.4) kg/m^2^. The study subjects were divided into a training set (*n* = 875, 70.0%) and a validation set (*n* = 375, 30.0%); no statistical difference was observed in all of the baseline characteristics between the two groups (all *p* > 0.05) (Table 1).

### 3.2. Univariate Analysis of Characteristic Variables for NAFLD in HTN Patients 

In the training set, univariate analysis revealed that statistically significant differences were observed in the following variables between NAFLD and non-NAFLD groups (all *p* < 0.05): region (OR = 0.615, 95% CI: 0.411–0.921); DBP (OR = 1.012, 95% CI: 1.000–1.024); BMI (OR = 1.176, 95% CI: 1.123–1.231); OADs (OR = 3.355, 95% CI: 2.361–4.768); AHs (OR = 4.635, 95% CI: 2.988–7.192); LLDs (OR = 2.407, 95% CI: 1.655–3.499); BUN (OR = 0.933, 95% CI: 0.881–0.988); TC (OR = 1.193, 95% CI: 1.058–1.345); TGs (OR = 1.433, 95% CI: 1.270–1.617); HDL-C (OR = 0.306, 95% CI: 0.183–0.513); LDL-C (OR = 1.156, 95% CI: 1.005–1.329); ALT (OR = 1.025, 95% CI: 1.013–1.038); AST (OR = 1.018, 95% CI: 1.000–1.035); AAR (OR = 0.347, 95% CI: 0.238–0.505); and WBC (OR = 1.082, 95% CI: 1.007–1.163) (Table 2).

### 3.3. Screening of Characteristic Variables via LASSO Analysis 

**Figure 2:**
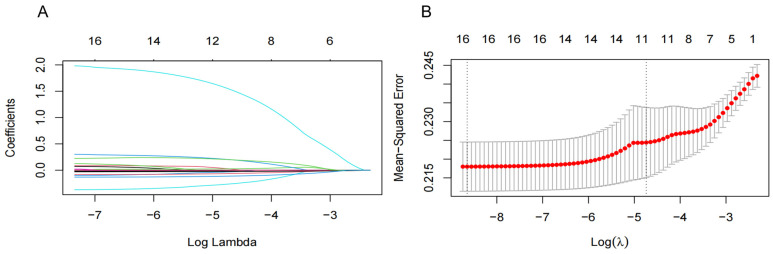
Screening significant variables for hypertension (**HTN**) patients with nonalcoholic fatty liver disease (**NAFLD**) via a Lasso binary logistic regression model. (**A**) Coefficient curves were generated for 16 clinical features. These 16 clinical features include the following: diastolic blood pressure (DBP); body mass index (BMI); region; type 2 diabetes mellitus (T2DM); oral antidiabetic drugs (OADs); antihypertensives (AHs); lipid-lowering drugs (LLDs); blood urea nitrogen (BUN); total cholesterol (TC); total triglyceride (TGs); high-density lipoprotein cholesterol (HDL-C); low-density lipoprotein cholesterol (LDL-C); alanine aminotransferase (ALT); aspartate aminotransferase (AST); and AST/ALT ratio (AAR). (**B**) Lasso regression was employed in a 10-fold cross-validation to select the most appropriate clinical features. The Lasso regression model was utilized for variable screening, selecting 16 non-zero variables by determining the optimal value in (**A**). The effective contour is then generated based on the optimal parameter in (**B**), including region and T2DM, OADs, AHs, LLDs, DBP, BMI, BUN, TC, TGs, HDL-C, LDL-C, ALT, AST, AAR, and WBC.

To improve the generalization and stability of the LASSO model for characteristic variables related to NAFLD, we included 15 significant variables in the univariate analysis and T2DM as independent variables and used tenfold cross-validation with the regularization parameter (λ) corresponding to the mean squared error (MSE) minimum plus one standard error (SE). The results showed that 16 variables with the optimal λ of 0.020, including region, T2DM, OADs, AHs, LLDs, DBP, BMI, BUN, TC, TGs, HDL-C, LDL-C, ALT, AST, AAR, and WBC, were selected as NAFLD-associated characteristic variables for the subsequent analysis (Figure 2).

### 3.4. Multivariate Analysis of Characteristic Variables for NAFLD in HTN Patients 

We next put HTN concomitant with NAFLD into the dependent variable (0 = No, 1 = Yes) and included 16 significant variables from the LASSO regression analysis as independent variables. Multivariate logistic regression analysis identified OADs (OR = 2.553, 95% CI: 1.368–4.768), AHs (OR = 7.303, 95% CI: 4.168–12.794), BMI (OR = 1.145, 95% CI: 1.084–1.209), TGs (OR = 1.474, 95% CI: 1.201–1.809), and AST (OR = 1.061, 95% CI: 1.018–1.105) as risk factors for HTN concomitant with NAFLD. Conversely, BUN (OR = 0.924, 95% CI: 0.860–0.992) and AAR (OR = 0.249, 95% CI: 0.121–0.514) were identified as protective factors for HTN concomitant with NAFLD (Table 3). Based on the results of the multivariate logistic regression analysis, the NAFLD risk-prediction model equation in HTN patients was constructed as follows:

Logit (P) = 0.937 × OADs + 1.988 × AHs + 0.135 × BMI − 0.080 × BUN + 0.388 × TGs + 0.059 × AST − 1.389 × AAR

**Figure 3:**
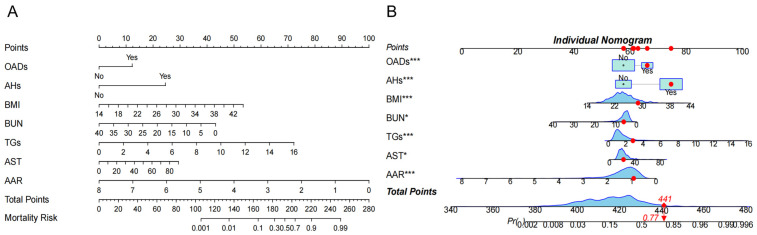
The risk nomogram of hypertension (HTN) patients with nonalcoholic fatty liver disease (NAFLD). (**A**). The nomogram model included oral antidiabetic drugs (OADs); antihypertensives (AHs); body mass index (BMI); blood urea nitrogen (BUN); total triglycerides (TGs); aspartate aminotransferase (AST), and AST/ALT ratio (AAR) for analysis. Each variable is assigned a specific score, and the total score from all variables corresponds to the probability of developing NAFLD risk. (**B**) One patient with HTN was randomly selected whose NAFLD incidence was predicted based on the seven characteristic indicators of the nomogram. The ne patient condition is shown on the graph and marked with a red dot, with the red diamond square representing the patient’s risk score after all variables have been summed, and the arrow pointing to the patient’s corresponding final risk probability of developing NAFLD. The symbol (*) next to a variable indicates the weight of its contribution to the total score, not statistical significance.

The above factors were further successfully constructed in the nomogram model. Each variable is assigned a specific score, and the total number of scores for all variables corresponds to the probability of developing NAFLD risk (Figure 3A). For example, suppose a patient with HTN has the following characteristics: BMI 28.73 kg/m^2^, BUN 6.15 mmol/L, TGs 2.81 mmol/L, AST 34 U/L, and AAR 0.917. This produces a score of 441, corresponding to a probability of 0.77, indicating a 77.0% risk of the HTN patient developing NAFLD (Figure 3B).

### 3.5. Model Evaluation and Comparison

**Figure 4:**
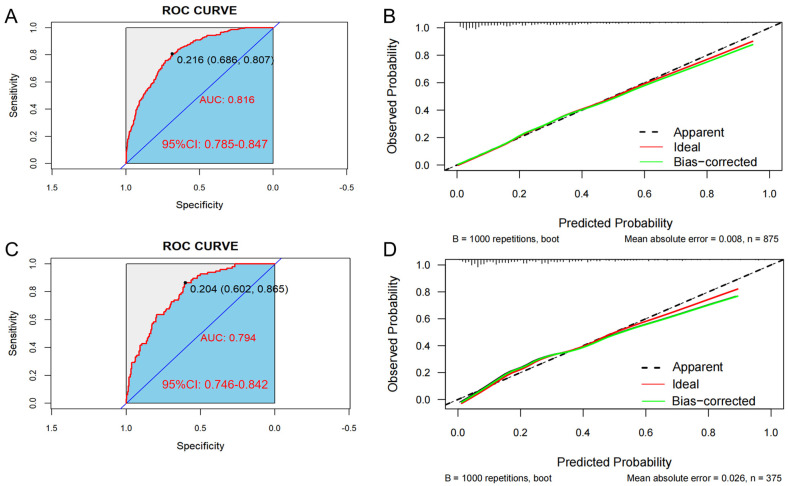
The area under the ROC curve (AUC) is used to evaluate the performance of the predictive model for hypertension (HTN) combined with nonalcoholic fatty liver disease (NAFLD). The *X*-axis represents the false positive rate predicted by the model; the *Y*-axis represents the true positive rate predicted by the model; the red curve represents the performance of the nomogram. Graphs represent the ROC curve of the model in the training set (**A**) and the validation set (**C**). Calibration curve of the risk of HTN patients with NAFLD: the *X*-axis of the curve represents the predicted risk of concurrent hypertension in patients with NAFLD; the *Y*-axis represents the actual diagnosed morbidity; the diagonal line represents the perfect prediction of the ideal model, and the higher overlap of the dark dashed line with the diagonal line represents the better accuracy of the model. Graphs represent the analysis of the calibration curve of the hypertension risk nomogram in the training set (**B**) and the validation set (**D**).

In the training set, the AUC was 0.816 (95% CI: 0.785–0.847), and the C-index was 0.807. The optimal critical value of the model was determined to be 0.204; the sensitivity and the specificity were 80.7% and 68.6%, respectively (Figure 4A). Additionally, in the validation set, the AUC was 0.794 (95% CI: 0.746–0.842), and the C-index was 0.776. The optimal critical value of the model was determined to be 0.216; the sensitivity and the specificity were 86.5% and 60.2%, respectively (Figure 4C). These results indicate that the predictive model demonstrates good discrimination in both the training and validation groups.

Bootstrap verification results for the calibration curves of the training and validation sets show that the absolute errors between the simulated and actual curves are 0.008 and 0.026, respectively, suggesting that the trends of the two curves are essentially consistent (Figure 4B,D). In addition, the Hosmer–Lemeshow test indicated that no significant difference between the predicted value and the real value was observed in both the training set (χ^2^ = 6.313, *p* = 0.612) and the validation set (χ^2^ = 10.672, *p* = 0.221), indicating the model has high calibration. 

### 3.6. Clinical Practicability of the Model

**Figure 5:**
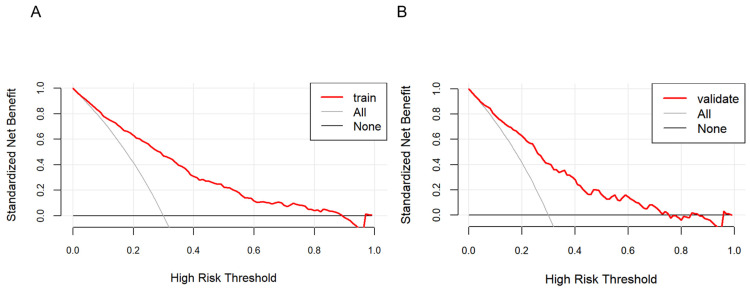
Decision curve analysis of the nonalcoholic fatty liver disease (NAFLD) risk nomogram in hypertension (HTN) patients. The solid line parallel to the horizontal axis in the graph indicates that when all samples are negative, the net benefit of having no intervention for all is zero. Backlash shows that all samples are positive and have received intervention, and the net benefit is the magnitude of the slope. The red line represents the net benefit of the model constructed in this study. The red line is from the two intersecting lines, the higher the clinical application value of the model. Graphs represent the decision curve analysis in the training set (**A**) and the validation set (**B**).

As shown in Figure 5, the farther the red line is from the two intersecting lines, the greater the net benefit of the constructed model, indicating a higher clinical application value of the model. DCA shows that the threshold probability of a patient is greater than 1%, suggesting the net benefit of using a nomogram to predict the risk of HTN patients concomitant with NAFLD is higher. 

## 4. Discussion

Based on the prediction model, BMI, OADs, AHs, TGs, and AST were identified as risk factors for HTN patients with NAFLD, while BUN and AAR were protective factors for HTN patients with NAFLD.

BMI is an important risk factor for HTN, especially in the prospective study by Chen et al., which included 2785 patients with HTN. Their risk prediction model included variables such as age, BMI, SBP, DBP, and fasting glucose [16]. BMI is a widely used measure of body fat. Obesity, especially central obesity (i.e., excess abdominal fat), is widely recognized as a major risk factor for HTN. Our study showed that BMI was an independent and significant risk factor for patients with NAFLD, with each unit increase in BMI increasing the risk of developing NAFLD with HTN by approximately 14.5%. This is consistent with the findings of previous studies. Studies have shown a significant association between BMI and fatty liver disease (including nonalcoholic fatty liver disease), the incidence of which increases with BMI [17,18]. NAFLD is closely associated with obesity; most NAFLD patients have obesity problems, and the degree of obesity is significantly correlated with the severity of NASH and liver fibrosis [19]. Controlling BMI, for example through weight management and improving body composition (e.g., increasing muscle mass, etc.), can effectively prevent or reduce the risk of HTN and NAFLD. A healthy diet, moderate physical activity, and lifestyle management are important ways to achieve ideal body weight and BMI levels that contribute to overall physical health.

The results of this study suggest that the use of OADs may be associated with the development or exacerbation of NAFLD. This may indicate that certain medications have adverse effects on the liver or that users of these medications have other underlying liver problems themselves. A possible reason for this is that certain OADs may cause an increased accumulation of fat in the liver by altering lipid metabolism or affecting liver cell function. Previous studies have shown that OADs can help improve NAFLD [20] but the conclusions of this study are inconsistent with those of previous studies. Although previous studies have shown that some glucose-lowering drugs, such as GLP-1 receptor agonists with SGLT-2 inhibitors, can reduce the incidence of NAFLD [21,22], and SGLT-2i even significantly reduces hepatic fat content and improves hepatic function [23,24], and thiazolidinediones (e.g., pioglitazone) also reduce the risk of NAFLD by enhancing insulin sensitivity (HR = 0.66, 95% CI 0.55–0.78) [20,25], the results of the present study differ from this. This contradiction may stem from the following factors: first, the present study did not differentiate between drug subclasses, whereas insulin and sulfonylureas may exacerbate hepatic lipid deposition through weight gain and hyperinsulinemia [26], and their risk effects may mask the potential benefits of newer medications; and second, it is worth noting that the association may have been confounded by an indication bias: patients requiring intensive glucose-lowering therapy typically have a longer diabetic duration, poorer glycemic control, and higher baseline metabolic risk, which are themselves strongly associated with NAFLD progression. In addition, drugs such as metformin, although not directly improving NASH histology, may indirectly affect hepatic fat metabolism in specific populations through weight loss [27]. The insufficiently large sample size, lack of data on drug subtypes and dosage, and residual confounders (e.g., visceral obesity, genetic background) in this study may limit the extrapolation of conclusive results. Future prospective cohort studies with large samples, combined with longitudinal liver imaging and refined drug exposure data, are needed to further analyze the class–effect relationship of specific hypoglycemic agents and the characteristics of high-risk populations. In conclusion, the association between antihyperglycemic drugs and NAFLD is highly heterogeneous, and individualized regimens based on individual metabolic characteristics and drug-specific mechanisms are needed in clinical practice.

Similarly, this study found that the use of AHs was associated with NAFLD. AHs may indirectly affect liver health by altering blood pressure regulation mechanisms or by affecting renal function. HTN is closely associated with NAFLD, and their interaction may exacerbate liver damage. Antihypertensive therapy, especially the use of ACE inhibitors, ARBs, and diuretics, may have different effects on patients with NAFLD. Studies have shown that ACE inhibitors and ARBs may have a protective effect on the liver by inhibiting the angiotensin-aldosterone system, reducing oxidative stress, and improving blood flow, which may help to slow the process of liver fibrosis [28,29].

However, the use of diuretics, especially thiazides and tab diuretics, may exacerbate the course of NAFLD by affecting electrolyte balance and increasing hyperglycemia and hyperuricemia [30,31]. Monotherapy is usually effective in controlling blood pressure, but combination therapy, especially when ACE inhibitors/ARBs are used in combination with diuretics, may increase the risk of side effects and exacerbate liver and kidney burden [32].

Therefore, in patients with NAFLD, the selection of AHs needs to be cautious, and it is recommended to individualize the treatment according to the patient’s specific situation and to monitor the liver function and electrolyte levels for a long time to ensure the safety and efficacy of the treatment. Future studies should further clarify the mechanism of action of different classes of antihypertensive drugs in NAFLD patients and their safety.

The results of this study showed that lipid-lowering drugs (LLDs) did not play a role in HTN patients with NAFLD. Previous studies have shown that the effect of LLDs on NAFLD is not clear [1]. However, in an observational study involving 11,593,409 Korean subjects, researchers found that statins showed some potential to reduce the risk of NAFLD and improve liver fibrosis [33].

This finding needs to be discussed and analyzed from multiple perspectives. First, the nature of observational studies means that the results can be influenced by a variety of potential factors, including confounding factors that are not fully controlled for, information bias, and limitations in inferring causation.

In addition, the results of this study show that BUN is a protective factor for NAFLD, and the risk of HTN patients with NAFLD is reduced by 0.027 times for each unit increase in BUN. Increased BUN levels may reduce the risk of HTN patients with NAFLD. Possible reasons are the specific role of BUN in liver metabolism or its interaction with other biomarkers, which may make it play a relatively protective role in the environment in which HTN patients with NAFLD co-occur. In addition, BUN is an indicator of liver function, and its level can be affected by a variety of factors, including nutritional status, kidney function status, and the complex effects of disease. In the context of the combination of HTN patients with NAFLD, decreased BUN levels may reflect improvements in liver function or changes in metabolic pathways that may reduce the risk of developing NAFLD. 

Notably, the protective effect of BUN may be potentially influenced by age and pharmacologic interventions. Previous studies have demonstrated a significant threshold effect on the incidence of liver-related events in patients with NAFLD at or above 50 years of age [34]. Additional studies have shown that BUN levels are significantly higher in the elderly population than in the younger group [35]. However, the negative correlation of BUN with age (independent of creatinine) in elderly patients with chronic kidney disease (CKD) suggests that the effect of age on BUN may be subject to complex nonlinear associations [31]. Thus, although the protective effect of BUN in the present study remained significant after correction for age, age-related fluctuations in BUN may partially explain the heterogeneity of the high-risk population.

On the other hand, the modulatory effects of antihypertensive drugs on BUN need to be further explored. For example, ACEI/ARBs may reduce BUN accumulation by improving renal hemodynamics [29,36], whereas diuretics (e.g., hydrochlorothiazide) may indirectly increase BUN levels due to decreased blood volume or glomerular filtration rate [29]. Dose optimization for combinations of medications (e.g., ACEI + diuretics) may partially offset the effect of a single medication on BUN [37]. However, the present study did not specifically subdivide the effect of specific drug types, and subgroup analyses or drug stratification models are still needed to test this hypothesis. The protective effect of BUN may be partially masked by confounding factors if the type of drug or dose is not adequately controlled for.

Previous studies on BUN as a protective factor for NAFLD were relatively few. BUN levels reflect the ability of the liver to synthesize urea. There is no direct evidence that BUN can be used as a protective factor for NAFLD, but some studies have explored that BUN may be indirectly related to liver health when renal function and metabolic status are stable [37,38,39]. Future prospective cohort or mechanistic studies are needed to clarify whether the protective effect of BUN is independent of age, pharmacologic interventions, and co-morbid states, and to explore the molecular pathways underlying it.

TGs serve as a crucial biochemical marker in blood, commonly utilized for evaluating lipid metabolism and the severity of fatty liver. The findings of this study demonstrate that elevated TG levels pose a significant risk for patients with NAFLD and T2DM, with each unit increase in TGs associated with an approximately 47.4% heightened risk. This underscores the substantial association between TGs and NAFLD occurrence in HTN patients, even when accounting for other influencing factors. Elevated TG levels often indicate aberrant fat metabolism, potentially leading to hepatic fat accumulation and thereby elevating NAFLD risk. Recognizing the potential of TGs as a predictive indicator for NAFLD holds significance for clinical practice and patient management, enabling early identification and intervention in high-risk individuals to mitigate NAFLD incidence and its complications. Several observational studies have implicated dyslipidemia as an independent risk factor for severe NAFLD-related liver conditions such as cirrhosis, cirrhosis-related complications, or liver-related mortality [40]. Epidemiological evidence has established high triglyceride levels as an independent risk factor contributing to both the onset and progression of NAFLD [41,42]. Elevated TG levels reflect disrupted fat metabolism, encompassing hepatic fat deposition and increased fatty acid synthesis—crucial pathways underlying NAFLD development [43].

In addition, high TG levels are strongly associated with an increased risk of progression to NASH, liver fibrosis, and cirrhosis in NAFLD patients. These developments not only affect the prognosis of patients but also increase the risk of liver complications such as liver cancer [44]. TGs may also be associated with increased all-cause and CVD-related mortality in NAFLD patients [45].

AST and ALT serve as crucial markers for assessing hepatic injury in individuals diagnosed with NAFLD, where their levels frequently exhibit elevation [46,47,48]. The use of the AAR as an indicator for identifying fatty liver disease has been documented; it typically presents at values below 1 among NAFLD patients [47,48,49,50]. Our investigation revealed that an AAR below 1 was linked to heightened risks associated with both high blood pressure and nonalcoholic fatty liver disease development—potentially influenced by aberrant hepatic function dynamics alongside inflammatory responses or metabolic statuses impacting the balance between AST and ALT ratios—a finding congruent with prior research outcomes [51,52]. Furthermore, our findings suggest that elevated levels of AST pose a risk factor when coupled with NAFLD, hinting at a marginal positive association between higher concentrations of AST and escalated risks possibly attributed to adipose tissue accumulation inducing mild inflammation causing hepatocellular impairment, thereby elevating enzyme release into circulation; whereas an elevated level exceeding 1 indicates augmented likelihoods for developing NAFLD within HTN cases, potentially reflecting extensive hepatic damage or underlying pathophysiological mechanisms driving its progression.

Moreover, given our cross-sectional design, certain inherent limitations might have influenced our findings, thus requiring cautious interpretation; therefore, vigilant monitoring is warranted, particularly among individuals presenting AAR values below 1, with a focus on regular surveillance specifically related to serum levels, including both AST and ALT. Frequent assessments could assist in early detection, facilitating timely interventions while emphasizing targeted educational initiatives aimed at enhancing awareness regarding hepatic well-being among high-risk cohorts, emphasizing lifestyle modifications encompassing balanced dietary habits, reduced alcohol consumption, maintenance of healthy body weights, and regular physical activity. All these pivotal measures not only assist in hypertension management but also foster improvement against progressive manifestations arising from NAFLDs while promoting optimal hepatological care.

### Strength and Limitation

In this study, we constructed a column-line graph prediction model of HTN with NAFLD based on the Guangxi population, and the model assessed the efficacy well, which filled the gap in the study of hypertension with NAFLD in this region. Nevertheless, there are some limitations in this study, which is a fact-finding study and can only derive the influencing factors of HTN combined with NAFLD, with some limitations on the interpretation of causality. There may be other influencing factors that could not be explored in this study, and deeper exploration is needed in the future. 

Future research should prioritize the following: ① prospective validation in multiethnic cohorts with serial measurements of dynamic indicators (e.g., weight trajectory, medication adjustments) to assess temporal associations; ② translational development of simplified risk scoring tools (e.g., open-access Shiny APP calculators) to enhance clinical adoption; and ③ mechanistic exploration via Mendelian randomization or real-world pharmacoepidemiologic analyses to disentangle drug–NAFLD interactions, particularly the protective role of emerging therapies like SGLT2 inhibitors.

## 5. Conclusions

Through binary logistic regression analysis, our study revealed that BMI, OADs, AHs, TGs, and AST were risk factors for HTN patients with NAFLD, whereas BUN and AAR were protective factors for HTN patients with NAFLD. The ROC curve and Hosmer–Lemeshow test indicated that the diagnostic specificity of the nomogram model was high. The DCA results demonstrated that this model has certain clinical practicability. 

In summary, the nomogram risk prediction model constructed in this study has a good discriminative ability for the occurrence of HTN patients with NAFLD. Medical workers can refer to this model for the early identification of high-risk groups among HTN patients with NAFLD and guide the formulation of targeted treatment and intervention measures.

## Figures and Tables

**Table 1:** Basic demographic characteristics of the study population.

Variable	Total (*n* = 1250)	Training Set (*n* = 875)	Validation Set (*n* = 375)	Statistic (Z/χ^2^)	*p*
Age (years)	66.0 (56.0, 73.0)	66.0 (56.0, 72.0)	67.0 (57.0, 73.0)	0.581	0.561
SBP (mmHg)	140 (130.0, 155.0)	139.0 (130.5, 155.0)	140.0 (130.0, 155.0)	0.335	0.738
DBP (mmHg)	84.0 (77.0, 92.0)	84.00 (76.0, 92.0)	84.00 (77.0, 93.0)	0.576	0.565
BMI (kg/m^2^)	24.2 (22.2, 26.4)	24.2 (22.2, 26.4)	24.2 (22.2, 26.3)	0.250	0.803
NAFLD, *n* (%)	303 (24.2)	207 (23.7)	96 (24.2)	0.540	0.463
Region, *n* (%)				0.357	0.550
Nanning	957 (76.6)	674 (77.0)	283 (75.5)		
Yulin	293 (23.4)	201 (23.0)	92 (24.5)		
Genders				1.461	0.227
Male	502 (40.2)	361 (41.3)	141 (37.6)		
Female, *n* (%)	748 (59.8)	514 (58.7)	234 (62.4)		
Nation, *n* (%)				0.390	0.532
Han ethnicity	1137 (91.0)	793 (90.6)	344 (91.7)		
Others	113 (9.0)	82 (9.4)	31 (8.3)		
Smoking, *n* (%)				0.023	0.989
Current smoker	65 (5.2)	45 (5.1)	20 (5.3)		
T2DM, *n* (%)	547 (43.8)	384 (43.9)	163 (43.5)	0.019	0.891
OADs, *n* (%)				0.114	0.736
Yes	254 (20.3)	180 (20.6)	74 (19.7)		
AHs, *n* (%)				0.262	0.609
Yes	837 (67.0)	582 (66.5)	255 (68.0)		
LLDs, *n* (%)				0.022	0.883
Yes	217 (17.4)	151 (17.3)	66 (17.6)		
BUN (mmol/L)	5.7 (4.5, 6.9)	5.53 (4.4, 6.9)	5.64 (4.6, 6.9)	1.201	0.230
Crea (mmol/L)	79.0 (64.0, 98.0)	79 (64, 98)	80 (65, 98)	0.531	0.595
TC (mmol/L)	4.6 (3.9, 5.4)	4.56 (3.85, 5.4)	4.68 (3.93, 5.51)	1.707	0.088
TGs (mmol/L)	1.8 (1.0, 2.1)	1.34 (0.97, 1.98)	1.44 (1.05, 2.17)	2.309	0.021
HDL-C (mmol/L)	1.2 (1.0, 1.4)	1.17 (0.98, 1.42)	1.15 (0.98, 1.41)	0.241	0.810
LDL-C (mmol/L)	2.8 (2.1, 3.5)	2.75 (2.11, 3.48)	2.81 (2.19, 3.50)	1.060	0.289
TBIL (μmol/L)	9.0 (6.6, 12.3)	9.1 (6.7, 12.4)	8.8 (6.4, 12.2)	1.066	0.286
ALT (U/L)	16.0 (12.0, 23.0)	16 (12, 23)	16 (12, 24)	0.302	0.763
AST (U/L)	20.0 (16.0, 25.0)	20 (16, 25)	20 (16, 26)	0.171	0.864
AAR	1.216 (0.941, 1.556)	1.2 (0.941, 1.556)	1.231 (0.941, 1.55)	0.135	0.898
WBC (10^9^/L)	6.745 (5.76, 8.08)	6.79 (5.73, 8.07)	6.73 (5.82, 8.120)	0.073	0.942

The continuous variables with skewed distribution are presented as medians (quartiles), and categorical variables indicate the number (percentage). The *p*-value was calculated by Pearson’s Chi-squared test or the Wilcoxon rank sum test. SBP: systolic blood pressure; DBP: diastolic blood pressure; BMI: body mass index; NAFLD: nonalcoholic fatty liver disease; OADs: oral antidiabetic drugs; AHs: antihypertensives; LLDs: lipid-lowering drugs; BUN: blood urea nitrogen; Crea: creatinine; TC: total cholesterol; TGs: triglycerides; HDL-C: high-density lipoprotein cholesterol; LDL-C: low-density lipoprotein cholesterol; TBIL: total bilirubin; ALT: alanine aminotransferase; AST: aspartate aminotransferase; and AAR: AST/ALT ratio.

**Table 2:** Univariate logistic regression analysis in HTN patients with NAFLD.

Variables	β	Odd Ratio	95% CI	*p*-Value
Region				
Nanning				1.00 (Reference)
Yulin	−0.486	0.615	0.411–0.921	<0.018
DBP (mmHg)	0.012	1.012	1.000–1.024	0.046
BMI (kg/m^2^)	0.162	1.176	1.123–1.231	<0.001
T2DM	0.31	1.364	0.998–1.865	0.052
OADs				
NO				1.00 (Reference)
YES	1.211	3.355	2.361–4.768	<0.001
AHs				
NO				1.00 (Reference)
YES	1.534	4.635	2.988–7.192	<0.001
LLDs				
NO				1.00 (Reference)
YES	0.878	2.407	1.655–3.499	<0.001
BUN (mmol/L)	−0.069	0.933	0.881–0.988	0.018
TC (mmol/L)	0.176	1.193	1.058–1.345	0.004
TGs (mmol/L)	0.359	1.433	1.27–1.617	<0.001
HDL-C (mmol/L)	−1.183	0.306	0.183–0.513	<0.001
LDL-C (mmol/L)	0.145	1.156	1.005–1.329	0.043
ALT (U/L)	0.025	1.025	1.013–1.038	<0.001
AST (U/L)	0.017	1.018	1.000–1.035	0.047
AAR	−1.059	0.347	0.238–0.505	<0.001
WBC (10^9^/L)	0.079	1.082	1.007–1.163	0.031

DBP: diastolic blood pressure; BMI: body mass index; OADs: oral antidiabetic drugs; AHs: antihypertensives; LLDs: lipid-lowering drugs; BUN: blood urea nitrogen; TC: total cholesterol; TGs: total triglyceride; HDL-C: high-density lipoprotein cholesterol; LDL-C: low- density lipoprotein cholesterol; ALT: alanine aminotransferase; AST: aspartate aminotransferase; and AAR: AST/ALT ratio.

**Table 3:** Logistic regression analysis of multiple factors and NAFLD in patients with HTN.

Variables	β	Odd Ratio	95% CI	*p*-Value
Intercept	−5.847	<0.05		0.001
OADs				
NO				1.00 (Reference)
YES	0.937	2.553	1.368–4.763	0.003
AHs				
NO				1.00 (Reference)
YES	1.988	7.303	4.168–12.794	<0.001
BMI (kg/m^2^)	0.135	1.145	1.084–1.209	<0.001
BUN (mmol/L)	−0.080	0.924	0.860–0.992	0.030
TGs (mmol/L)	0.388	1.474	1.201–1.809	<0.001
AST (U/L)	0.059	1.061	1.018–1.105	0.005
AAR	−1.389	0.249	0.121, 0.514	<0.001

BMI: body mass index; OADs: oral antidiabetic drugs; AHs: antihypertensives; BUN: blood urea nitrogen; TGs: triglycerides; AST: aspartate aminotransferase; and AAR: AST/ALT ratio.

## Data Availability

All data generated or analyzed in this study are included in this published article. Due to non-public data, the datasets generated and/or analyzed in this study are not publicly available but may be obtained from the corresponding authors upon reasonable request.
